# Ocular Characteristics of Patients with Leber Congenital Amaurosis 6 Caused by Pathogenic *RPGRIP1* Gene Variation in a Chinese Cohort

**DOI:** 10.1155/2021/9966427

**Published:** 2021-11-09

**Authors:** Yumei Mao, Yanling Long, Bo Liu, Qingling Cao, Yijian Li, Sha Li, Gang Wang, Xiaohong Meng, Shiying Li

**Affiliations:** ^1^Ophthalmology Department, Southwest Hospital, Army Medical University (Third Military Medical University), Chongqing, China; ^2^Key Lab of Visual Damage and Regeneration and Restoration of Chongqing, Chongqing, China; ^3^Department of Ophthalmology, The Second Affiliated Hospital of Chongqing Medical University, Chongqing 400010, China; ^4^Department of Ophthalmology, Xiang'an Hospital of Xiamen University, Medical Center of Xiamen University, School of Medicine, Xiamen University, Xiamen, China; ^5^Eye Institute of Xiamen University, Xiamen, China

## Abstract

**Purpose:**

To delineate the clinical and genetic characteristics of Chinese patients with *RPGRIP1*-associated Leber congenital amaurosis 6 (LCA6).

**Methods:**

After screening 352 unrelated families with clinically diagnosed RP, five LCA6 patients with *RPGRIP1* variations from unrelated Chinese families were identified. Full ophthalmology examinations, including decimal best-corrected visual acuity (BCVA), fundus photography, fundus autofluorescence imaging, spectral-domain optical coherence tomography (SD-OCT), full-field electroretinography (ffERG), multifocal electroretinography (mfERG), perimetry, and flash visual evoked potential (FVEP), were performed. Target next-generation sequencing (NGS) and Sanger sequencing were performed for the five patients to identify and to validate candidate disease-causing variants.

**Results:**

Five patients were molecularly diagnosed as the LCA6 associated with *RPGRIP1* variation, with typical clinical characteristics including congenital night blindness, nystagmus, and visual defect, at an early age. Interestingly, LCA6 exhibited extensive clinical heterogeneity and the changes in the morphology and function were not completely consistent in the five LCA6 patients. Case 1 showed extensive inferior-nasal retinal atrophy with a corresponding area of hypofluorescence in fundus autofluorescence, and the fundus photograph was nearly normal in cases 2 and 3. The ERG results displayed a moderately reduced rod-system response in cases 1 and 2 and a significant reduced rod-system response in case 3. Both case 4 and case 5 showed mottled pigmentation in fundi and an unrecordable rod and cone-system response in ERG. Moreover, we identified eight compound variants and one homozygous variant in the five patients with *RPGRIP1*.

**Conclusions:**

This is the largest report focused on the clinical electrophysiological features of patients with associated LCA6 caused by the variation in the *RPGRIP1* gene in the Chinese population with an enriched phenotypic and genotypic background of LCA6 to improve future gene therapies.

## 1. Introduction

Leber congenital amaurosis (LCA, MIM 204000) is a group of severe and early onset inherited retinal disorders (IRDs) with a worldwide prevalence of 1/33000 [1]∼1/81000 [2] and contributes to approximately 10%∼20% of blind children in school [1]. LCA is a highly clinically and genetically heterogeneous disease characterized by blindness and pendular nystagmus within the first year of life with a later development into photoreceptor death with serious vision loss, pigmentary retinopathy, and minimal or nondetectable electroretinogram (ERG) responses. Currently, 25 genes (*CRX, IMPDH1, OTX2, AIPL1, CABP4, CCT2, CEP290, CLUAP1, CRB1, DTHD1, GDF6, GUCY2D, IFT140, IQCB1, KCNJ13, LCA5, LRAT, NMNAT1, PRPH2, RD3, RDH12, RPE65, RPGRIP1, SPATA7,* and *TULP1*) with autosomal recessive or dominant inheritance have been identified to be related to LCA (https://sph.uth.edu/Retnet/, accessed July 14, 2021).

Among them, Leber congenital amaurosis 6 (LCA6, MIM 613826) is caused by variations in the *RPGRIP1* gene encoding retinitis pigmentosa GTPase regulator interacting protein 1 (RPGRIP1), which participates in ciliary transport processes. LCA6 is inherited as an autosomal recessive trait and is estimated to account for 6% of patients with LCA in America [3] and 8.8% of LCA cases in China [4]. LCA6 patients present with a stable and nonprogressive disease course after the initial rapid decline in visual function, and the morphology of photoreceptors in the central retina can persist for a long time. Thus, LCA6 associated with *RPGRIP1* gene variants are expected to be treated by a gene replacement strategy [5, 6]; however, despite the latest advances in gene therapy on LCA2 (*RPE65*) [7] and LCA10 (*CEP290*) [8], there is no available clinically applicable treatment for LCA6 at present.

It is important to objectively assess the fundus morphology and visual pathway of LCA6 patients as well as the application of a visual function evaluation after treatment in the future. In this study, we aimed to delineate the genotype and phenotype in five unrelated Chinese LCA families with identified *RPGRIP1* variants by targeting next-generation sequencing (NGS) technologies.

## 2. Methods

### 2.1. Patient Recruitment

Five patients from five unrelated families with a clinical diagnosis of LCA6 were screened from 352 unrelated families with inherited retinal degeneration at the Ophthalmology Department, Southwest Hospital, Army Medical University, Chongqing, China, from 2014 to 2018 and were retrospectively included in this study [9]. Informed consent was obtained from each subject prior to proceeding with the examinations. The study was approved by the local ethics committee (reference number: KY2020096), and all procedures were performed in accordance with the principles of the Declaration of Helsinki. A detailed family history was obtained through interviews with the patients and their relatives.

### 2.2. Clinical Examinations

The ophthalmic examination, including decimal best-corrected visual acuity (BCVA) measurements, fundus photography (Kowa, Tokyo, Japan), fundus autofluorescence imaging (FAF, Heidelberg Engineering, Heidelberg, Germany), perimetry, spectral-domain optical coherence tomography (SD-OCT; Heidelberg Engineering, Heidelberg, Germany), perimetry (HFA7501, Zeiss, Germany), flash visual evoked potential (FVEP; Roland Consult, Germany), multifocal electroretinography (mfERG; EDI, San Mateo, CA), and full-field electroretinography (ffERG; Diagnosys LLC, Lowell, MA), were performed according to the standard protocol of the International Society for Clinical Electrophysiology of Vision (ISCEV). All patients underwent a comprehensive ERG examination that included the following: (1) dark-adapted dim flash 0.01 candela second (cd.s)/m^2^ (dark-adapted 0.01), (2) dark-adapted bright flash 3.0 (cd s)/m^2^ (dark-adapted 3.0), (3) dark-adapted bright flash 10.0 (cd s)/m^2^ (dark-adapted 10.0), (4) light-adapted 3.0 (cd s)/m^2^, and (5) light-adapted 30 Hz flicker ERG (light-adapted 30 Hz).

### 2.3. Molecular Genetic Analysis Using NGS

Genomic DNA extraction, targeted next-generation sequencing, and bioinformatics analysis were followed by the methods of Dr. Meng et al. 2021 [10]. Targeted next-generation sequencing (NGS) was performed using a capture panel including 195 known inherited retinal disease (IRD) genes (Supplementary Table 1). The clinical phenotypic findings and cosegregation analysis (by Sanger sequencing) were fully considered when we identified the disease-causing variants of patients.

## 3. Results

### 3.1. Clinical Findings

**Figure 1:**
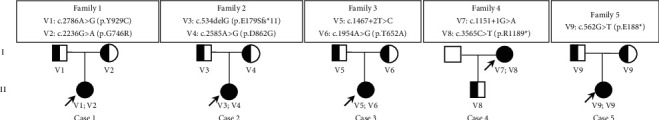
Pedigrees of Chinese families with LCA6 harboring pathogenic *RPGRIP1* variants. The proband is indicated by an arrow. The squares and circles indicate men and women, respectively. The black solid square or circle represents the affected individual. The half black solid circle or square represents the variation carrier. V: variant.

Five affected subjects from five unrelated families with a diagnosis of LCA6 and who harbored *RPGRIP1* variants were identified (Figure 1). The clinical features are summarized in Table 1.

**Figure 2:**
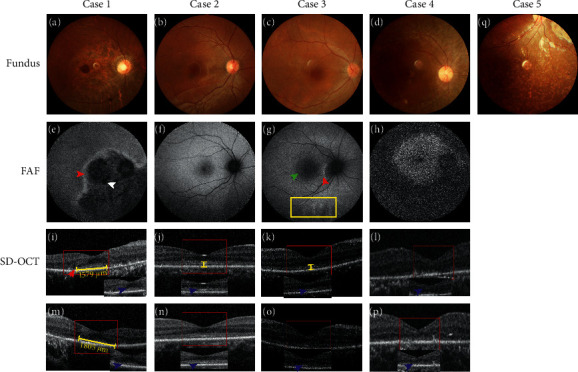
Fundus photography, fundus autofluorescence imaging, and foveal optical coherence tomography scans of probands from the LCA6 families. The fundus image of both eyes in case 1 (a: right eye), the pigment mottling pattern, symmetry atrophy of the retina at the inferior-nasal area, and AF of the right eye (e: right eye). Hypofluorescence is coincident with an area of the retina atrophy, mottled pigmentary (white narrow), and hyperfluorescent boundary (red narrow). The SD-OCT scan showed that the ellipsoid zone is preserved in the central macular that marks the horizontal length with a yellow line, 1579 *μ*m and 1805 *μ*m, respectively (blue arrows; i: right eye, m: left eye). The boundary line of the ellipsoid zone (red arrow) between the reserved and atrophy areas (i: right eye, m: left eye). Fundus photographs (b right eye) and autofluorescence (f: right eye) of case 2 indicate both eyes are close to normal, while the SD-OCT of both eyes (j: right eye, n: left eye) shows that the adjacent lamellar structure is thinner and that there is a preserved ellipsoid zone within a 6 mm horizontal length (blue narrows, yellow line). The case 3 fundus (c: right eye) does not show an abnormality, but the enlarged hypofluorescence area of the macula (green arrow), hyperfluorescent ring (red arrow) around the optic disc, and the subtemporal mottled hyperfluorescence (yellow box) (g: right eye). The OCT shows the thinning of all retinal layers and blurred ellipsoid areas (blue arrows and yellow line; k: right eye, o: left eye). The fundus image of case 4, peripheral pigmentation, and the pigment change (d: right eye). The AF of the right eye shows that except for a concentric hyperfluorescent area (hyperfluorescent ring), it is coincident with an area of no retinal pigmentation and pigmentary deposits in the remaining area of the retina (h: right eye). The OCT shows that both eyes of all layers are unrecordable, including the disappearing ellipsoid zone in the foveal area (blue arrows; l: right eye, p: left eye). The fundus photographs in case 5 show attenuated retinal vessels and a mottled pigmentary change (q: right eye).

The patients were all female and exhibited congenital night blindness, nystagmus, and severe visual impairment in their infancies. The age at disease diagnosis ranged from 5 to 34 years, and the clinical course of LCA6 displayed a different sequence of events among the five probands. Case 4 and case 5 had the worst visual acuity, only light perception, and HM. Their fundus appearances were also similar, and they exhibited retinal atrophy with intraretinal vascular attenuation, a waxy disc, and the pigment mottling pattern (Figures 2(d) and 2(q)). Accordingly, the fundus autofluorescence revealed an area of a hyper-autofluorescence ring in the para-foveal region in case 4 (Figures 2(h)), and her SD-OCT images (Figures 2(l) and 2(p)) revealed a worsened preservation of adjacent lamellar structures in which retinal pigment epithelium (RPE) and photoreceptor cells showed different degrees of loss in the central macula. Moreover, the visual field of these two patients was too poor to measure and the ERG responses of DA and LA were unrecordable.

The BVCA of patient case 3 was only 0.05 in both eyes, the hypofluorescence area of the macula was enlarged, with a hyperfluorescent ring around the optic disc and the subtemporal mottled hyperfluorescence, and her fundus photographs and were similar to case 2. Case 1 and case 2 showed both 0.15 OD and 0.15 OS. The fundus photographs were grossly normal in case 2 (Figures 2(b) and 2(f)) and case 3 (Figures 2(c) and 2(g)), while case 1 exhibited mottled pigmentary and depigmentation, vascular attenuation, and extensive inferior-nasal retinal atrophy with a corresponding area of hypofluorescence (Figures 2(a) and 2(e)). The SD-OCT images revealed a thinner photoreceptor outer nuclear layer (ONL) with a preserved ellipsoid zone in case 2 (Figures 2(j) and 2(n)) and case 3 (Figures (2(i), 2(k), 2(m), and 2(o)). In addition, the horizontal length of the remaining ellipsoid zone of case 1 was about 1579 *μ*m in the right eye and 1805 *μ*m in the left eye (Figures (2(i), 2(k), 2(m), and 2(o)). The ellipsoid zones of case 2 and case 3 were observed at a horizontal length of 6 mm.

The visual field test indicated symmetry defects in superior-temporal vision fields as observed on the hypofluorescence in the right eye of case 1 (Supplemental Figure 1A), a tubular visual field in case 3 (Supplemental figure 1C), and a small center visual island defect in case 2 (Supplemental figure 1B). The visual fields of the remaining two patients were too poor to measure.

**Figure 3:**
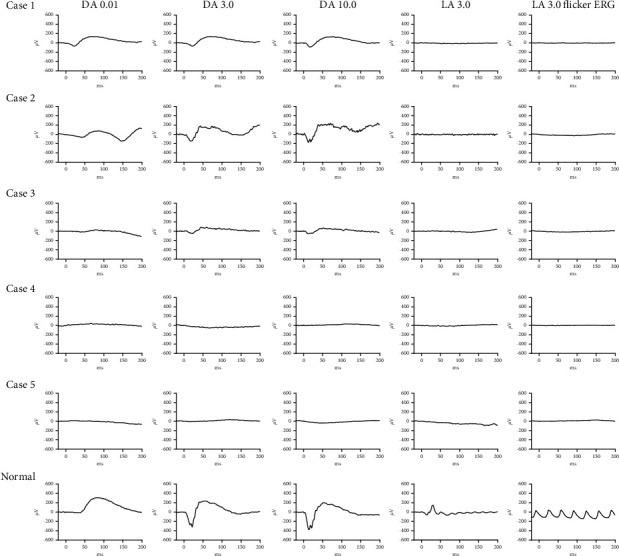
Full-field ERG recordings in patients with LCA6. Case 1, case 2, and case 3 all showed a moderate to severe loss of the rod-system response and an extinguished cone response, but case 3 had a worsened rod response compared to the other two. The rod responses and cone responses were nondetectable for case 4 and case 5. The last row is the normal control.

The mfERG revealed that the density of the wave amplitude had significantly declined in the right eyes of cases 1 and 2 (Supplemental Figure 1D and 1E) and the densities of the other three were too poor to measure. The ERG amplitudes of the a- and b-waves of patients with LCA6 were different compared with the normal control, and the differences were classified into two groups: Group 1 was assessed by the ERG response of DA as more than almost a quarter of the normal control and included case 1 and case 2 (Figure 3). Group 2 was defined by an ERG response of DA of less than almost a quarter of the normal control and included case 3, case 4, and case 5 (Figure 3). The response of the cone (LA 3.0 and LA 3.0 flicker ERG) was unrecordable for all five patients (Figure 3). Case 5 showed a severely reduced amplitude of all waves in the FVEP response, especially the amplitude of P2, which was worse than case 1 and case 2 (Supplemental Figure 2), but the patient's visual function was still assessed. All the basic symptoms supported the clinical diagnosis of LCA6. However, it is interesting that there was an inconsistency in the morphology and function among case 1, case 2, and case 3. The function was similar, but the morphology was different for case 1 and case 2. Furthermore, the morphology was the same, but the function was different for case 2 and case 3. And, the clinical symptom of case 4 and case 5 was typical LCA6.

### 3.2. Molecular Genetic Findings

We screened for variants of the five affected patients from five unrelated and nonconsanguineous families with known IRD-associated genes using NGS data (Table 2). Nine variants of the *RPGRIP1* gene were identified and were consistent with the intrafamilial cosegregation analysis, including four missense variants: c.1954A > G (p.T652A), c.2236G > A (p.G746R), c.2585A > G (p.D862G), and c.2786A > G (p.Y929C); two nonsense variants: c.562G > T (p.E188^*∗*^) and c.3565C > T (p.R1189^*∗*^); two splicing variants: c.1151+1G > A and c.1467 + 2T > C; and one frameshift variant: c.534delG (p.E179Sfs^*∗*^11). Except for the homozygous variant p.E188^*∗*^ in family 5, all other variants exist in a compound heterozygous state. Variant p.G746R in exon 15 [11] and p.R1189^*∗*^ in exon 22 [12] have been previously reported to be pathogenic variants of LCA in the compound heterozygous state, and the remaining seven variants have never been reported in ClinVar or HGMD Professional. Moreover, the allele frequency for four *RPGRIP1* variants (p.E179Sfs^*∗*^11, p.G746R, c.1151+1G > A, and p.R1189^*∗*^) were extremely low at 0.001441%, 0.0008747%, 0.0004091%, and 0.002007% in gnomAD, respectively. The remaining five *RPGRIP1* variants were absent from the controls in the 1000 genome, gnomAD, or EXAC databases. According to the American College of Medical Genetics and Genomics guidelines, the five variants, p.E179Sfs^*∗*^11, p.E188^*∗*^, c.1151+1G > A, p.R1189^*∗*^, and c.1467 + 2T > C, were assessed to be “pathogenic,” the variants, p.G746R, p.T652A, and p.D862G, were considered to be “likely pathogenic,” and the variant p.Y929C was of “uncertain significance.”

## 4. Discussion

*RPGRIP1* has also been reported to be associated with cone-rod dystrophy 13 (CROD 13), in which the phenotype of patients seemed to have been characterized by a rapid loss of vision in the second decade of life, but further details were not provided [13]. The clinical distinction between LCA and CORD is not clear, and there are large overlaps in the phenotype. In the present study, we reported the detailed clinical and genetic characteristics of five unrelated patients who were diagnosed with LCA6 caused by variation of the *RPGRIP1* gene. All five patients exhibited nystagmus and visual loss at or soon after birth, and case 2 and case 3 had similar morphological characteristics in the fundus photographs and OCT. Case 2 belonged to group 1, and case 3 belonged to group 2 for the ERGs. Furthermore, both case 1 and case 2 belonged to group 1 for the electrophysiological response, while they had a different fundus appearance and fundus autofluorescence, even for the OCT. According to the literature, the fundus appearance of patients with *RPGRIP1* associated with LCA is highly variable, and the fundus photographs have mentioned disc pallor, attenuated vessels, the pigment epithelium mottling, or bone spicules from the mid-retina to the periphery [14–18], sometimes with a grossly normal appearance [3, 16, 18]. The funduscopy of our patients with LCA6 has been reported as mentioned above, but some different information which was symmetry atrophy of the retina with mottled pigmentary at the inferior-nasal side had been discovered in case 1. There have been no reports regarding fundus photographs until now. Other researchers have only reported the high variability of fundus appearance, and there are no reports on *RPGRIP1*-related LCA6 patients' functions. Thus, we assessed the patient's local and overall visual function in detail, such as perimetry, mfERG, ffERG, and FVEP. We found that there was an inconsistency in the morphology and function among case 1, case 2, and case 3. Therefore, different examination methods can reflect the patient's progress and the morphological and functional differences of different patients with the same disease. Due to their different principles and characteristics, various types of examinations had significance to our subsequent clinical diagnoses. The fundus manifestations and clinical symptoms of case 4 and case 5 were typical LCA6.

The OCT indicated that the lamellar structure in the foveal area is retained to some extents from case 1 to case 3, with the exception of case 4. Blue arrows revealed that the ellipsoid zone was partially retained in case 1, a large area was preserved for case 2, and the ellipsoid zone was blurred in case 3; however, the ellipsoid zone disappeared in case 4. A recent study showed that all patients' phenotypes appeared to have a thinning of the photoreceptor outer nuclear layer (ONL) and that a retinal lamellar structure was maintained, whereas the ellipsoid zone was blurred or disappeared [18]. In addition, Suzuki et al. and Imani et al. described *RPGRIP1*-related LCA patients as having a loss of a normal foveal configuration, which is similar to case 4 [16, 19]. The phenotypic variability was high. OCT can only reflect the macular area, and it cannot reflect the morphology or function in other areas of the retina. Therefore, it is necessary to diagnose different types of hereditary retinopathy using different examination methods, which involves partial or overall phenotypes between the morphology and function.

The *RPGRIP1* gene is located on chromosome 14q11 and contains 24 exons. It encodes a protein which expresses in the cilium, connecting the outer and inner segments of human photoreceptor [20, 21]. A previous study revealed that mice lacking *RPGRIP1* showed high defects in disc formation photoreceptor outer segments and photoreceptor loss at 20 days and 5 months of age, respectively [5]. Moreover, a better preservation of photoreceptor function in the treated eyes after gene therapy on a murine model with *RPGRIP1* variants has been observed by an ERG and histological examination [6]. Lhériteau et al. provided great promise for human treatment: gene addition therapy can restore the functional deficit of cone photoreceptors and prevent the retinal degeneration and vision loss in *RPGRIP1*-deficient dogs, a model exhibiting a severe cone-rod dystrophy similar to that seen in humans [22]. Overall, *RPGRIP1* is indispensable in the disc morphogenesis of photoreceptors. To date, in HGMD Professional, 87 missense/nonsense variations in *RPGRIP1* have been reported. Among them, 27 (59%) of the 47 missense variations are located at the C2 domain and the following was RID, which occupied 6 (13%). This indicates that the C2 domain could likely be a hot spot for missense variants. In this study, we found that two novel missense variants (p.T652A and p.D862G) are associated with the C2 domain (Supplemental Figure 3). Moreover, the clinical symptoms of case 4 and case 5 were the most severe and may be related to the type of variation they carried, which was predicted to be loss-of-function variation. No genotype to phenotype correlation could be established in our cohort, but the disease-causing gene of *RPGRIP1* in these five LCA6 subjects diagnosed was rigorously cosegregated with the genotype and phenotype from 352 unrelated families with inherited retinal degeneration and expands the variant spectrum of *RPGRIP1*. Therefore, future studies with a larger sample size of LCA patients with *RPGRIP1* variants should be conducted using longitudinal clinical assessments and a genotype-phenotype correlation analysis.

In conclusion, we identified the heterogeneous retinal morphology changes and visual functions of *RPGRIP1*-LCA6 patients. The combination of perimetry, ffERG, FVEP, and other detection methods not only help in objectively assessing the fundus morphology and visual pathway of LCA6 patients but also will aid in the application of visual function evaluations after treatment in the future. We will conduct a large-scale screening LCA6 clinical cohort study among LCA patients in China to establish genotype to phenotype correlations to explore new treatments, such as gene therapy, as well as genetic counseling.

## Figures and Tables

**Table 1:** Clinical features of patients with LCA6.

Patient ID	Sex	Age		Initial symptoms	LogMARVA		Clinical features				
		Onset	Diagnosis		OD	OS	Visual field	SD-OCT	Autofluorescence	Fundus	FEGR
Case 1	F	1 y	34 y	PVADC	0.15	0.2	Binocular superior-temporal hemianopia	The ellipsoid zone preserved	Retinal atrophy with mild surrounding pigmentation	MPC	LNRR and ECR
Case 2	F	1 y	5 y	PVADC	0.15	0.15	A small center visual island defect	The ellipsoid zone preserved	Normal	Normal	LNRR and ECR
Case 3	F	1 y	28 y	PVADC	0.05	0.05	Tubular vision	The ellipsoid zone blurred	The increase of hypofluorescence area of the macula, hyperfluorescent ring, and mottled hyperfluorescence	Normal	LNRR and ECR
Case 4	F	<1 y	32 y	PVADC	HM	HM	N/A	All retinal layers are unclear	Concentric area of hypoautofluorescence	MPC	ECRR
Case 5	F	<1 y	9y	PVADC	LP	LP	N/A	NA	NA	MPC	ECRR

ECR: extinguished cone response; ECRR: extinguished cone and rod response; HM: hand movement; LNRR: lower than normal rod response; LP: light perception; NA: not available; PVADC: photophobia, visual acuity decreased, congenital nystagmus; MPC: mottled pigmentary change.

**Table 2:** The identified variants of *RPGRIP1*^#^ in Chinese patients with LCA6.

Patient	Exon	Nucleotide substitution	Amino acid change	Hom/Het	SIFT	PROVEAN	PolyPhen-2	MutationTaster	HSF Matrix	gnomAD	rs	Report	ACMG
*Case 1*	17	c.2786A > G	p.Y929C	Het	Damaging	Deleterious	Probably damaging	Disease causing	Alteration of an exonic ESE site, potential alteration of splicing	0	—	This study	US (PM2, PP1, PP3)
	15	c.2236G > A	p.G746R	Het	Damaging	Deleterious	Probably damaging	Disease causing	Activation of an exonic cryptic acceptor site, with presence of one or more cryptic branch point(s)	0.001441%	rs535695411	Reported	LP (PS1, PM1, PP1, PP3)

*Case 2*	4	c.534delG	p.E179Sfs^*∗*^11	Het	—	—	—	Disease causing	Alteration of an exonic ESE site, potential alteration of splicing	0.0008747%	—	This study	P (PVS1, PM4, PP1, PP3)
	16	c.2585A > G	p.D862G	Het	Damaging	Deleterious	Possibly damaging	Disease causing	Creation of an exonic ESS site, potential alteration of splicing	0	—	This study	LP (PM2, PM3, PP1, PP3)

*Case 3*	11	c.1467+2T > C	—	Het	—	—	—	Disease causing	Alteration of the WT donor site, most probably affecting splicing	0	—	This study	P (PVS1, PM2, PP3)
	14	c.1954A > G	p.T652A	Het	Damaging	Deleterious	Probably damaging	Disease causing	No impact	0	—	This study	LP (PM1, PM2, PM3, PP3)

*Case 4*	9	c.1151+1G > A	—	Het	—	—	—	Disease causing	Alteration of the WT donor site, most probably affecting splicing	0.0004091%	—	This study	P (PVS1, PM3, PP1, PP3)
	22	c.3565C > T	p.R1189^*∗*^	Het	—	—	—	Disease causing	Creation of an exonic ESS site, potential alteration of splicing	0.002007%	—	Reported	P (PVS1, PS1, PM4, PP1, PP3)

*Case 5*	4	c.562G > T	p.E188^*∗*^	Hom	—	—	—	Disease causing	Activation of an exonic cryptic donor site, potential alteration of splicing	0	—	This study	P (PVS1, PM2, PM4, PP1, PP3)

^#^The transcripts of the *RPGRIP1* we used in this study for sequencing and reference was NM_020366.4. LP: likely pathogenic; P: pathogenic; US: uncertain significance; -: not available.

## Data Availability

The data used to support the findings of this study are included within the article.

## Supplementary Materials

Supplementary Materials The supplementary material for this article can be found online. Supplemental Figure 1: visual field and mfEGR of cases 1, 2, and 3. Supplemental Figure 2: FVEP of cases 1, 2, and 5. Supplemental Figure 3: RPGRIP1 protein structure diagram and analysis of variation conservation. Supplementary Table 1: 195 inherited retinal disease genes analyzed by targeted NGS diagnostic testing.
