# Supplementary material for: Ocular Characteristics of Patients with Leber Congenital Amaurosis 6 Caused by Pathogenic RPGRIP1 Gene Variation in a Chinese Cohort
Source: J Ophthalmol. 2021 Nov 9;2021:9966427. doi: 10.1155/2021/9966427 (PMC8595035; doi:10.1155/2021/9966427)
Supplement: Supplementary Materials — The supplementary material for this article can be found online. Supplemental Figure 1: visual field and mfEGR of cases 1, 2, and 3. Supplemental Figure 2: FVEP of cases 1, 2, and 5. Supplemental Figure 3: RPGRIP1 protein structure diagram and analysis of variation conservation. Supplementary Table 1: 195 inherited retinal disease genes analyzed by targeted NGS diagnostic testing. [file 9966427.f1.docx]

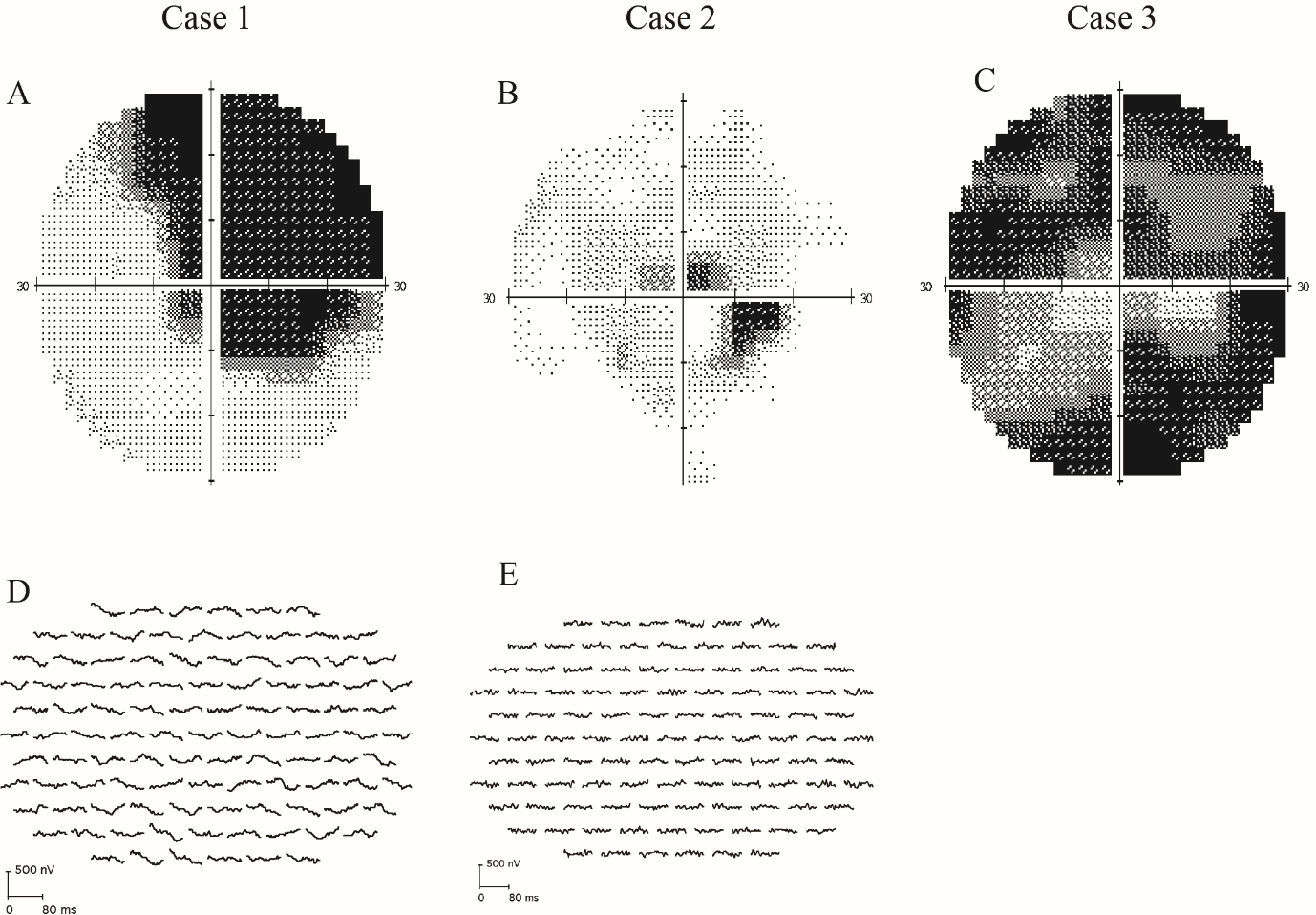


**Supplemental figure 1. Visual field and mfEGR of case 1, 2 and 3.**

The visual field of case 1 showed a symmetry defect at the superior-temporal retinal (A: right eye), and the mfERG exhibited decreased amplitude waves in all areas (D: right eye). For case 2, two eyes of the visual field displayed decreased central visual acuity (B: right eye), and the mfERG showed decreased amplitude waves (E: right eye). For case 3, the visual field displayed a tubular visual field (C: right eye).

**
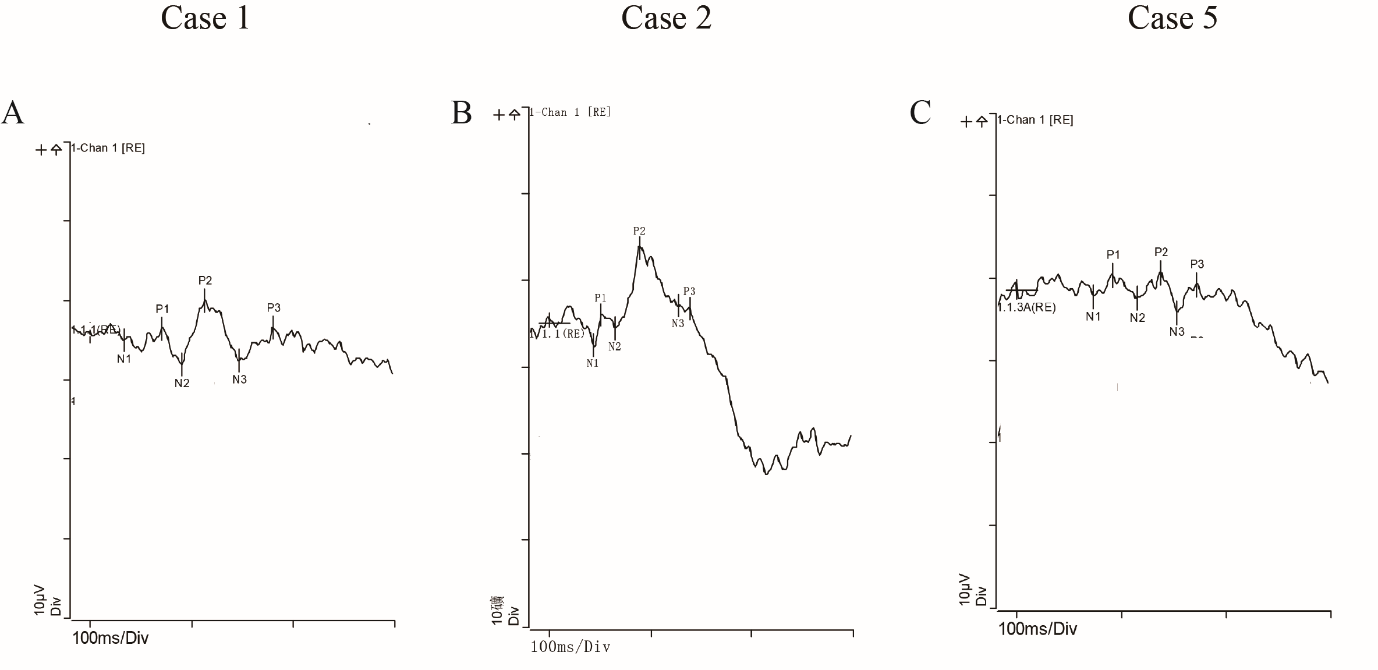
**

**Supplemental figure 2. FVEP of case 1, 2 and 5.**

The FVEP showed a P2 wave amplitude that was moderately decreased without a peak time delay in case 1 (A: right eye) and case 2 (B: right eye), while the FVEP of case 5 displayed a P2 wave with an amplitude that was severely decreased with peak time delayed (C: right eye).


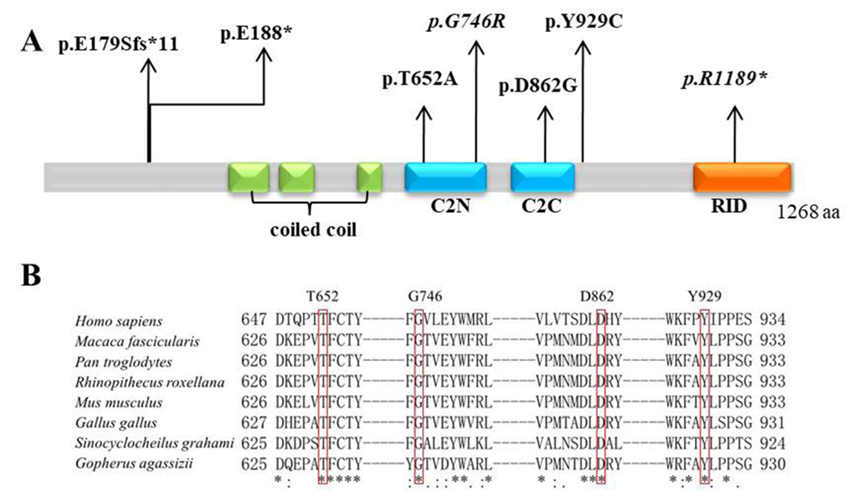


**Supplemental figure 3. RPGRIP1 protein structure diagram and analysis of variation conservation**

A: The positions of seven detected pathogenic variants (except splicing variants) in the RPGRIP1 protein. RPGRIP1 is a 1286-aa protein containing three N-terminal coiled coil domains (316-384aa, 403-462aa, and 537-578aa, respectively, highlighted in green), two C2 domains (C2N and C2C, 618-757aa and 799-908aa, respectively, highlighted in blue), and RID (RPGR-interacting domain, 1114-1279aa, highlighted in yellow) in the C-terminal (Reference: Pfam protein domain database; http://pfam.xfam.org/protein/Q96KN7). The positions of the seven detected pathogenic missense variants (arrow) in this study are shown. The previously reported variant is in italic style.

B: Multiple alignment of eight species of RPGRIP1. The alignment was performed with the Clustal Omega program, and the asterisk indicates a completely conserved residue in evolution. The positions of the mutated residues are highlighted by a red frame.

**Supplementary Table 1. 195 inherited retinal disease genes analyzed by targeted NGS diagnostic testing.**

| *ABCA4* | *BEST1* | *DHX38* | *IMPG1* | *NR2E3* | *RDH12* | *SNRNP200* |
| --- | --- | --- | --- | --- | --- | --- |
| *ABCB6* | *C1QTNF5* | *DRAM2* | *IMPG2* | *NRL* | *RDH5* | *SPATA7* |
| *ABCC6* | *C2orf71* | *EFEMP1* | *KCNJ13* | *OFD1* | *REEP6* | *SPP2* |
| *ADIPOR1* | *C5orf42* | *ELOVL4* | *KCNV2* | *OTX2* | *RGR* | *TIMP3* |
| *AGBL5* | *C8orf37* | *EMC1* | *KIAA1549* | *PDE6A* | *RHO* | *TMEM67* |
| *AHR* | *CA4* | *EYS* | *KIZ* | *PDE6B* | *RLBP1* | *TOPORS* |
| *ARHGEF18* | *CCDC28B* | *FAM161A* | *KLHL7* | *PDE6G* | *ROM1* | *TRNT1* |
| *ARL2BP* | *CEP290* | *FSCN2* | *LCA5* | *POMGNT1* | *RP1* | *TTC8* |
| *ARL3* | *CERKL* | *GNAT1* | *LRAT* | *PRCD* | *RP1L1* | *TTC8* |
| *ARL6* | *CFH* | *GPR125* | *LZTFL1* | *PRDM13* | *RP2* | *TULP1* |
| *BBIP1* | *CLCC1* | *GUCA1B* | *MAK* | *PROM1* | *RP9* | *USH1C* |
| *BBS1* | *CLRN1* | *HGSNAT* | *MERTK* | *PRPF3* | *RPE65* | *USH1G* |
| *BBS10* | *CNGA1* | *HK1* | *MFSD8* | *PRPF31* | *RPGR* | *USH2A* |
| *BBS12* | *CNGB1* | *HMCN1* | *MKKS* | *PRPF4* | *RPGRIP1* | *VCAN* |
| *BBS2* | *CRB1* | *IDH3B* | *MKS1* | *PRPF6* | *RPGRIP1L* | *WDPCP* |
| *BBS4* | *CRX* | *IFT140* | *MVK* | *PRPF8* | *SAG* | *ZNF408* |
| *BBS5* | *CTNNA1* | *IFT172* | *MYO7A* | *PRPH2* | *SAMD11* | *ZNF513* |
| *BBS7* | *CYP4V2* | *IFT27* | *NEK2* | *PTHB1* | *SEMA4A* | *CABP4* |
| *BBS9* | *DHDDS* | *IMPDH1* | *NEUROD1* | *RBP3* | *SLC7A14* | *CCT2* |
| *CLUAP1* | *DTHD1* | *GDF6* | *GUCY2D* | *IQCB1* | *NMNAT1* | *RD3* |
| *AIPL1* | *GUCA1A* | *PITPNM3* | *RIMS1* | *UNC119* | *ADAM9* | *ATF6* |
| *C21orf2* | *CACNA2D4* | *CDHR1* | *CEP78* | *CEP78* | *CNGA3* | *CNGB3* |
| *CNGB3* | *CNNM4* | *CNNM4* | *RS1* | *GNAT2* | *IFT81* | *PDE6C* |
| *PDE6H* | *POC1B* | *RAB28* | *RAX2* | *SLC4A7* | *TTLL5* | *CACNA1F* |
| *GNB3* | *GPR179* | *GRK1* | *GRM6* | *LRIT3* | *SLC24A1* | *TRPM1* |
| *NYX* | *TIMM8A* | *ABHD12* | *CDH23* | *CEP250* | *CIB2* | *CAPN5* |
| *FZD4* | *ITM2B* | *LRP5* | *RB1* | *TSPAN12* | *KIF3B* | *MAPKAPK3* |
| *RCBTB1* | *CDH3* | *OAT* | *OPN1LW* | *ASRGL1* | *RBP4* |  |
